# Population Movement and Virus Spreading: HEV Spreading in a Pilgrimage City, Mashhad in Northeast Iran; an Example

**DOI:** 10.5812/hepatmon.10255

**Published:** 2013-08-04

**Authors:** Sanaz Ahmadi Ghezeldasht, Rahele Miri, Mohamadreza Hedayatimoghadam, Aliakbar Shamsian, Hamidreza Bidkhori, Fahad Fathimoghadam, Seyyed Abdorrahim Rezaee

**Affiliations:** 1 Research Center for HIV/AIDS, HTLV and Viral Hepatitis, Iranian Academic Center for Education, Culture and Research (ACECR), Mashhad, IR Iran; 2 Academic Center for Education, Culture and Research (ACECR), Mashhad, IR Iran; 3 Immunology Research Center, Mashhad University of Medical Sciences, Mashhad, IR Iran

**Keywords:** Hepatitis E Virus, Population, Viruses

## Abstract

**Background:**

Hepatitis E Virus (HEV) infection is a significant public health concern and responsible for large outbreaks of acute hepatitis in poor sanitary and living conditions.

**Objectives:**

To investigate the impact of population movements on virus spreading, a large-scale population-based survey was performed in a pilgrimage- tourism area, the great Mashhad, capital city of Khorasan province.

**Patients and Methods:**

A cross-sectional study was carried out among 1582 randomly selected individuals from general population of Mashhad, north east of Iran, between May to September 2009. Serum samples were tested for total anti-HEV antibody using a specific enzyme linked immunoassay (ELISA) kit.

**Results:**

The prevalence of HEV infection was 14.2% (225/1582) with a maximum of 25.5 % (14/55) in densely populated areas. The highest prevalence was observed in visitant areas (≥ 20%) near the holly shrine with crowded hotels and inns. The differences between these areas and other districts were statistically significant (P < 0.001). The findings indicated that 13.2% (95/718) of males and 15.0% (130/864) of females were HEV positive; this difference is not significant. Seroprevalence increases with age rising , from 12.8% in subjects less than five years to 28.6% in individuals with more than 65 years old. Although, there were no meaningful differences between HEV seropositivity and socio-economic status, Illiterate individuals were significantly at higher risk for infection than educated persons (P < 0.001).

**Conclusions:**

These findings demonstrated that, high prevalence of HEV is related to populated district, which can reach to the highest rate in hotels and inns close to visitants. Traditional sanitation and water supplying systems are the second important factor for the virus transmission. Therefore, it can be concluded that such areas need efficient surveillance systems to prevent the spreading of infectious diseases.

## 1. Background

Epidemiological studies have shown that hepatitis E virus (HEV) infection is a significant public health concern in many parts of the world, causing large outbreaks of acute hepatitis. This virus was first recognized in the early 1980s (1) and known as the causative agent of hepatitis E. HEV is a small, non-enveloped, single-stranded RNA virus, approximately 27-34 nm in diameter. In most recent ICTV (International Committee on Taxonomy of Viruses) classification, HEV has been placed in its own taxonomic group "Hepatitis E-like viruses" as a member of the genus Hepevirus in the Hepeviridae family (2), within the class IV positive sense RNA viruses (3). The viral particles are relatively stable in the environment and have been recovered from sewage samples (4). Since the first documented hepatitis E outbreak in India during 1955–1956, there have been many large outbreaks reported in developing countries of Southeast and Central Asia, the Middle East, northern and western parts of Africa (5-9). In contrast, in developed countries, there have been sporadic cases of locally acquired hepatitis E, even though; no epidemics have been reported (10, 11). Although the overall mortality rate associated with HEV infection is low, it is reportedly as high as 20% among infected pregnant women particularly during the third trimester of pregnancy (12, 13),children younger than two years old, (14, 15) organ recipients, (16, 17) other severely immunocompromised subjects (18) and in blood products recipients (19). The infection has been primarily described to be associated with the ingestion of fecally contaminated drinking water as a waterborne disease (12). However, recent investigations have not consistently found well-defined water sources of HEV, suggesting other possible routes of transmission (14, 20). These other transmission modes may be related to the level of population immunity, sanitary conditions, living conditions and other factors (21). Other frequent routes of transmission have been demonstrated such as blood transfusion and person to person (22). It is also considerable that, in some populations, HEV appears to be easily transmissible with up to 76% of people aged >20 years old having serological evidence of infection without any significant disease (23). Where hepatitis E is hyperendemic or not, the frequency of various transmission routes, the affected groups and disease characteristics differ sufficiently in different areas.

The HEV genotypes prevalent in regions with different patterns of disease epidemiology vary and these may determine some of the differences in disease epidemiology in these regions (24). Mashhad as the main city in north east of Iran and the capital city of Khorasan, is a populated holly city for Muslims which is located near the geographical border of Afghanistan and Turkmenistan. The motives of mobility, the process of the international movement, particularly those from in countries at war with poor sanitary conditions such as Iraq and Afghanistan (25, 26) and the back and forth transition between differential risk environments should be considered for management of HEV infection in immigrant-receiving metropolitan areas such as Mashhad. Moreover, the main source of water distribution either piped line or other water supply systems, for drinking and household needs, during drought since 2000 in the region, have been the underground water in the city. On this period, sewage is disposed in traditional way and was not connected to the local network of urban sewage disposal system.

Therefore, pilgrimage, immigration, tourism and traditional sewage system may alter the epidemiology of HEV infection from other parts of the country. To our knowledge, few studies have addressed the prevalence of HEV infection in pilgrimage and tourism region, particularly in Iran. The current study was conducted to investigate the sero-epidemiology of HEV among general population in this region that have a huge mobile population and accepted immigrants and asylum seekers, mostly from Iraq and Afghanistan. 

## 2. Objective

Hepatitis E Virus (HEV) infection is found worldwide and is responsible for large outbreaks in East and South Asia. To investigate the impact of population movement on virus spreading, a large-scale population-based survey was performed in a pilgrimage-tourism area, the great Mashhad, capital city of Khorasan province.

## 3. Patients and Methods

This analysis is a cross-sectional study that was conducted in 2012 in the Academic Center for Education, Culture and Research (ACECR) Mashhad branch, Iran. Blood samples were taken after obtaining an informed consent and the sera were stored at -20 until test performance. The source survey of this study was performed on the general population of Mashhad the great, between May and September 2009. The subjects were selected randomly by multistage sampling methods (stratified, cluster, and systematic) from all of twelve municipality areas and forty districts of the city. In each household, one person was selected so that we tried to include equal ratios of the both sexes as well as ten percentiles for the age according to the 2006 census in each district.

Whole blood sample were collected and sera were assessed for total anti-HEV antibody using a commercial specific enzyme-linked immunosorbant assay (ELISA) kit (Diapro, Italy) according to the manufacturer's instruction. Data were analyzed with SPSS software using Chi-square, fisher and T-test. Statistical significance was set at P < 0.05. A multivariate logistic regression analysis was performed to identify variables that were independently associated with presence of anti-HEV.

## 4. Results

### 4.1. Seroprevalence of HEV

Total of 1582 were assayed for HEV total antibody. The mean age was 29.06 ± 18.513 years, range from one to 90 years old, 718 (45.38%) were male and 864 (54.62%) were female. The overall seroprevalence of HEV in this population was 14.2% (225 /1582).

Furthermore, 3.0 % (47/1582) of the whole study population were in the range of equivocal results. The finding indicated that 13.2% (95/718) of males and 15.0% (130/864) of female were HEV positive; however, the difference was not statistically significant. Furthermore as, Table 1 shows that the seroprevalence of HEV increased significantly with rising the age, from 12.8% in subjects less than five years to 28.6% in individuals with 65 years old and over. Therefore, a higher prevalence of anti-HEV positivity was seen in older subjects (P < 0.001). 

**Table 1.** The Age-Specific Prevalence of Anti-HEV

Age group	HEV infection			Total Count (Within Age Group), No. (%)
	Negative Count (Within Age Group), No. (%)	Equivocal Count (Within Age Group), No. (%)	Positive Count (Within Age Group), No. (%)	
**< 5**	75 (87.2)	0 (0.0)	11 (12.8)	86 (100.0)
**5-14**	240 (89.2)	5 (1.9)	24 (8.9)	269 (100.0)
**15-24**	364 (89.7)	8 (2.0)	34 (8.4)	406 (100.0)
**25-34**	253 (84.6)	12 (4.0)	34 (11.4)	299 (100.0)
**35-44**	147 (78.6)	5 (2.7)	35 (18.7)	187 (100.0)
**45-54**	113 (71.5)	9 (5.7)	36 (22.8)	158 (100.0)
**55-64**	69 (69.0)	2 (2.0)	29 (29.0)	100 (100.0)
**> = 65**	49 (63.6)	6 (7.8)	22 (28.6)	77 (100.0)
**< 5**	75 (87.2)	0 (0.0)	11 (12.8)	86 (100.0)

### 4.2. The Seroprevalence of HEV and Socio-Economic Status

There were not any statistically significant relations between HEV seropositivity and socioeconomic status such as income, marital status and mother’s educational level. However, there was a meaningful relation between illiteracy and HEV sero-prevalence (P < 0.001). Illiterate individuals were at higher risk of infection than educated persons (Table 2). 

**Table 2.** Literacy Level and HEV Infection

Literacy level	HEV infection			
	Negative Count (within literacy level)	Negative Count (within literacy level)	Negative Count (within literacy level)	Negative Count (within literacy level)
**Illiterate**	68 (64.2)	8 (7.5)	30 (28.3)	106 (100.0)
**Literate**				
Primary-Secondary School	495 (82.0)	16 (2.6)	93 (15.4)	604 (100.0)
High School-Pre university	413 (85.9)	16 (3.3)	52(10.8)	481 (100.0)
Academic	219 (84.9)	6 (2.3)	33 (12.8)	258 (100.0)

### 4.3. The Seroprevalence of HEV in Twelve Municipality Areas of Mashhad

**Figure 1.**
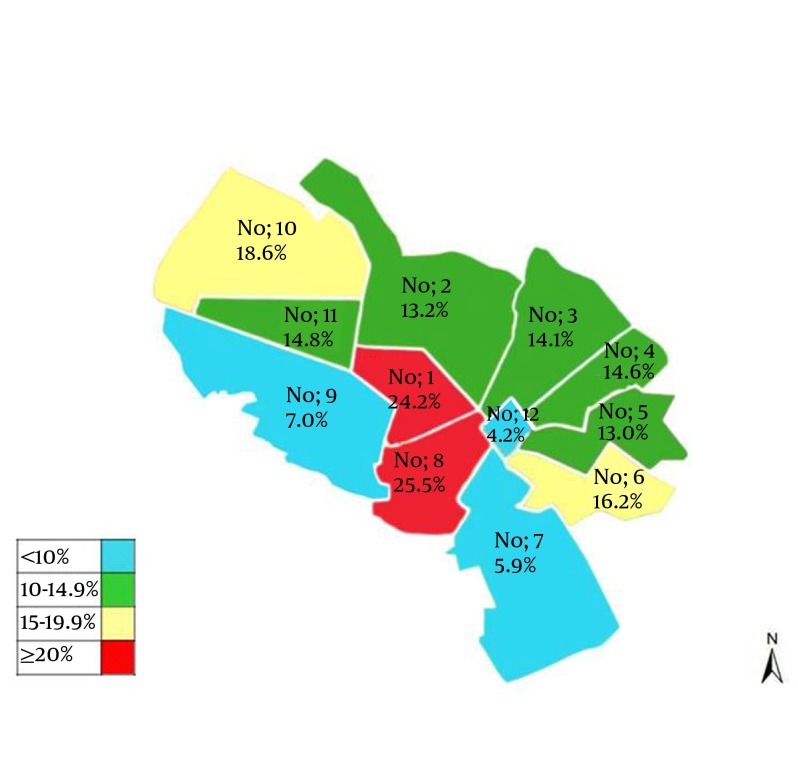
Distribution of HEV Prevalence in Twelve Municipality Areas of Mashhad

HEV antigen was considerably different among different districts in twelve municipality areas of Mashhad. As Figure 1 shows a significant difference (P < 0.001) was observed between HEV prevalence and the place of living in twelve different municipality areas of the city. HEV was detected in patients from almost all districts of Mashhad, including; the lowest rate in 12th district (4.2%), with the fewest Mashhadi residents, and the highest percentages in the 8th district (25.5%), a densely populated area (city center) with high density of hotels and inns.

## 5. Discussion

The present study is the largest population-based sero-epidemiological work of HEV infection in Iran and the first conducted in a pilgrimage tourism area in north east of the country. Hepatitis E is an important public health concern in many developing countries of Asia and Africa particularly drought and war stricken regions, where environmental sanitation facilities are poor, (27, 28). Large outbreaks of hepatitis E were observed in, India, Pakistan, Nepal, Myanmar, China and East Africa (29). Usually hepatitis E occurs in areas with a high population density, lowlands, and valley areas. The incidence and prevalence rate of HEV infection is underestimated due to unavailability of laboratory services in endemic territories. It is believed that in India at least one half of acute sporadic viral hepatitis cases are etiologically associated with HEV (30). Iran is located between Iraq and Turkey in the west, Afghanistan and Pakistan in the east on border.

Mashhad as the main city in north east of Iran is a holly city which is located near the geographical border of Afghanistan and Turkmenistan. Mashhad, with population of Three million people (census 2006), attracts more than 25 million tourists and pilgrims every year. Therefore, pilgrimage, immigration and tourism may alter the epidemiology of HEV infection compare to the whole country.

The results of study on the overall HEV prevalence were 14.2%, which is higher than several previous population-based studies and reports among general population in Iran (28, 31, 32). A report from general population in Mazandaran (North of Iran) revealed that 1.1% of children younger than 10 years and 7.2% of population between 20 and 25 years old were positive for anti-HEV IgG antibody (33). A study in Nahavand, a city in Hamadan province, has shown 9.3% HEV prevalence in general population (32). A report from Isfahan revealed that the overall anti-HEV seroprevalence was 3.8% in general population (34). The prevalence is even different in various cities in a certain population, which is 7.4% on hemodialysis patients in Tabriz (35) and 7% in Jahrom (36), 27.5% in East Azerbaijan on patients with chronic liver disease (37) and 11.5% in Khuzestan on Blood donor(28). Difference of HEV prevalence in the general population, the criteria for inclusion of samples, mobile populations, tourism and pilgrimage, sewage disposal and the routes of HEV transmission could partially explain the diverse results. The results of the present study is less than general population in Pakistan (17.5%) (38). There is only one study of outbreak in Afghanistan revealed that during 2004-2005, the prevalence of anti-HEV in veterans who have had their military service in Afghanistan was 29.97% (39). In Iran, the large cities have better public health services, such as clinics, municipal water and sewage systems, possibly explaining the reduced risk of infection; However, Mashhad as a popular pilgrimage city and in neighborhood with HEV high prevalence regions was accounted as an endemic city for hepatitis E. HEV seropositivity has been mainly reported in males (28, 40). However, most studies in Iran fail to demonstrate any significant differences between male and female (31, 33, 34, 37). The results of this study also confirmed which HEV infection is not significantly related to gender. The seroprevalence increased significantly with rising age (P < 0.001). Except two studies, (41, 42) the results of the most previous studies in Iran have shown that HEV seropositivity is (32, 34, 37, 43, 44) lower in educated populations compared to the uneducated. However, the differences were not significant (32, 34, 37, 43, 45). In the present study, the results supported a strong relationship between HEV seropositivity and illiteracy (P < 0.001).

Contaminated water is a potential source for the spread of this transmissible enteric disease. Pigs and animals have a minor role in HEV infection in our region. During drought, beginning from 2000, water resources in Mashhad has been turned to underground water. However, the sewage from the city, houses, and factories, is mainly disposed in traditional way and was not connected to the local network of urban sewage disposal system, mainly in city center. As Figure 1 shows, the high prevalence of HEV appears in district, No 1 and 8 municipalities of the city where sewage disposal system is currently (2010) connected to the water supply system of Iran–Turkmenistan Friendship Dam. 

**Figure 2.**
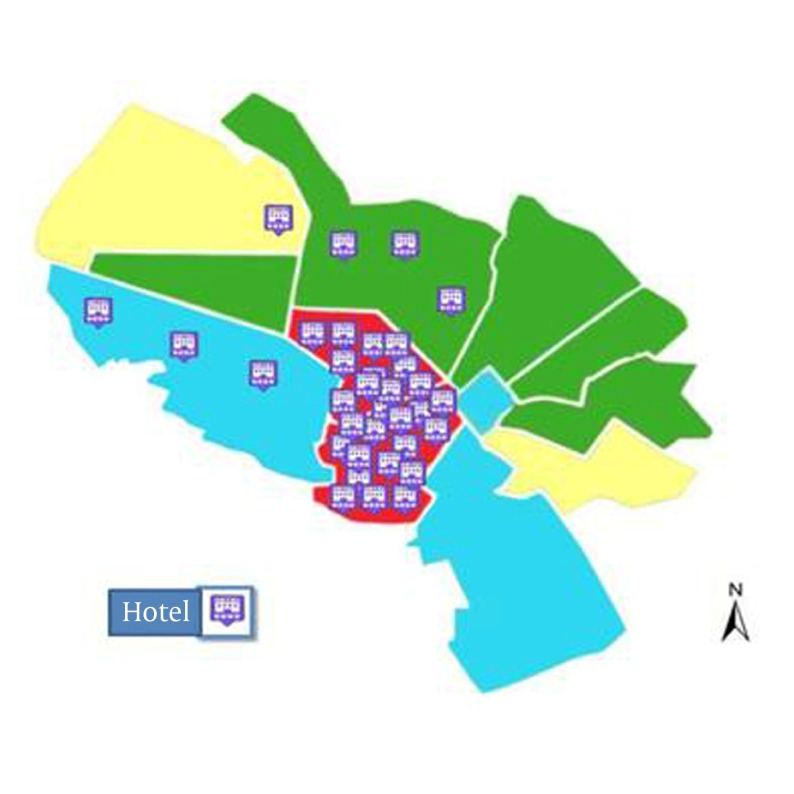
Distribution of Hotels in Mashhad

Usually hepatitis E occurs in areas with a high population density (44). The population density is not the main factor for virus spreading among population. Problems may rise when different mobile populations mixed up with the visitants from all parts of the world result in overcrowding. Population movement from HEV high prevalence areas to populated residential areas and overcrowding is happening during Muslim's holly months in Mashhad; hence, the rate of virus transmission should be very high. Thus, the high density of population in some municipality areas of the holly Mashhad, explains the high prevalence of HEV. As it was mentioned above, Mashhad has a mixed population of residents and travelers with close contact. Therefore, mobile population settled in hotels and inns in specified regions near holly shrine from different departures could easily spread the communicable infections. On the other hand, preparing healthy environment in such situation for residents is hardly achievable, due to the close contact between the visitants and unhealthy environment. As Figure 2 shows, these factors might result in the high prevalence of hepatitis E in those municipality areas of Mashhad. Migration and population mobility are associated with continuing and increasing infectious diseases such as HEV that could effectively be controlled if those factors are taken into consideration. 

Taken together, the poor maintenance of the sewage disposal system and the drinking water supply line from underground water, overcrowded area, unhealthy environment during pilgrimage seasons and emigration may have resulted in the high prevalence of HEV in Mashhad, which is even higher than war stricken areas in country such as Khuzestan (28). Prevention of hepatitis E relies primarily on the provision of clean water supplies, preparing sanitary water for agriculture, especially vegetables, avoiding contamination of water pipelines with sources of infection and establishing proper disposal systems. Furthermore, the results in the present study show that establishment of a good plan in order to prepare healthy environment for pilgrimage season in overcrowded areas could not only prevent spreading of viruses among residents of such big cities, but also eliminate the chance of spreading the infectious diseases, particularly hepatitis around the country. In addition, clinicians in such area should be aware and consider that the emergent of infections such as HEV, could be presented with unexplained signs and symptoms. In case of HEV, due to narrow window for diagnosis, prompt collection of samples for laboratory testing should be considered.
